# SELF- AND PROXY-REPORTED MET NEEDS IN PERSONS WITH DEMENTIA ARE ASSOCIATIED WITH A LOWER HEALTH-RELATED QUALITY OF LIFE

**DOI:** 10.1093/geroni/igad104.2557

**Published:** 2023-12-21

**Authors:** Joost Wammes, Holly Laws, Hein van Hout, Janet MacNeil Vroomen, Joan Monin

**Affiliations:** Amsterdam Medical Centers, Amsterdam, Noord-Holland, Netherlands; University of Massachusetts, Amherst, Amherst, Massachusetts, United States; Amsterdam Medical Centers, Amsterdam, Noord-Holland, Netherlands; Amsterdam Medical Centers, Amsterdam, Noord-Holland, Netherlands; Yale School of Public Health, New Haven, Connecticut, United States

## Abstract

This study examined the dyadic association of self and informal caregiver proxy-reported met needs in persons living with dementia on the health-related quality of life (HRQOL). A total of 237 dementia care dyads were included from the Case Management of Persons With Dementia and Their Caregivers Study. HRQOL was assessed by the EuroQol-5D and the number of met needs by the Camberwell Assessment of Needs for the Elderly. The Actor-Partner Interdependence Model framework was used to analyze the effect of an individual’s self or proxy-reported met needs on their own HRQOL (actor effects), and an individual’s self or proxy-reported met needs on the other dyad member’s HRQOL (partner effects). The number of self-reported met needs by persons living with dementia was negatively associated with their own HRQOL (actor effect b = -0.200, p < 0.001), and the HRQOL of informal caregivers (partner effect b = -0.114, p = 0.001). The number of proxy-reported met needs by informal caregivers was negatively associated with their own HRQOL (actor effect b = -0.105, p < 0.001) but not the person living with dementia’s HRQOL (-0.025, p = 0.375). Study findings suggest that both self-reported and informal caregiver proxy-reported met needs in persons living with dementia should be considered in research and practice because they have different implications for each dyad members’ HRQOL.

